# Comparison of the effectiveness of home visits and telephone follow-up on the self-efficacy of patients having undergone coronary artery bypass graft surgery (CABG) and the burden of their family caregivers: A randomized controlled trial*

**DOI:** 10.17533/udea.iee.v40n1e014

**Published:** 2022-03-30

**Authors:** Foruzan Gohari, Shirin Hasanvand, Mohammad Gholami, Heshmatolah Heydari, Parastoo Baharvand, Mohammad Almasian

**Affiliations:** 1 Student Research Center. Email:goharif52@yahoo.com Lorestan University of Medical Sciences, Khorramabad, Iran Lorestan University of Medical Sciences Khorramabad Iran goharif52@yahoo.com; 2 Associate Professor, Social Determinants of Health Research Center. Email: hasanvand.sh@lums.ac.ir. Lorestan University of Medical Sciences, Khorramabad, Iran Lorestan University of Medical Sciences Khorramabad Iran hasanvand.sh@lums.ac.ir; 3 Associate Professor. Email: mohammad13565@yahoo.com. Lorestan University of Medical Sciences, Khorramabad, Iran Lorestan University of Medical Sciences Khorramabad Iran mohammad13565@yahoo.com; 4 Associate Professor. Email: hidari74@gmail.com. Lorestan University of Medical Sciences, Khorramabad, Iran Lorestan University of Medical Sciences Khorramabad Iran hidari74@gmail.com; 5 Associate Professor. Email:Dr.baharvand@gmail.com Lorestan University of Medical Sciences, Khorramabad, Iran Lorestan University of Medical Sciences Khorramabad Iran Dr.baharvand@gmail.com; 6 Instructor. Email: almasian.m@lums.ac.ir Lorestan University of Medical Sciences, Khorramabad, Iran Lorestan University of Medical Sciences Khorramabad Iran almasian.m@lums.ac.ir

**Keywords:** self efficacy, coronary artery bypass, self-management, house calls, telenursing, caregivers., autoeficacia, puente de arteria coronaria, automanejo, visita domiciliaria, teleenfermería, cuidadores., autoeficácia, ponte de artéria coronária, autogestão, visita domiciliar, telenfermagem, cuidadores.

## Abstract

**Objective.:**

This study aimed to compare home visits and telephone follow-up effectiveness on patients' self-efficacy undergoing Coronary Artery Bypass Graft Surgery -CABG- and caregivers’ burden.

**Methods.:**

In this randomized clinical trial, 114 patients undergoing CABG were assigned to the three groups of home visits, telephone follow-up, and control based on the stratified block randomization. The self-management program of the home visit group included four face-to-face 60-minute training sessions once a week, and for the telephone follow-up group, four 30-minute telephone counseling sessions twice each week for a month. The control group received routine care. Data were collected using the cardiac rehabilitation self-efficacy questionnaire and the caregiver burden scale before and after the intervention.

**Results.:**

Before the study, there were no statistically significant differences between the three groups in terms of the means of self-efficacy and caregiver burden scores. However, there was a statistically significant difference between the home visit and control groups (*p*<0.001) and between the telephone follow-up and control groups (*p*<0.001) after the intervention, with increased self-efficacy and reduced caregiver burden reported. In contrast, there was no significant difference between the home visit and telephone follow-up groups regarding self-efficacy and caregiver burden scores.

**Conclusion.:**

Both methods of self-management education have similar effectiveness in increasing self-efficacy and reducing the caregiver burden after discharge for patients who have undergone CABG.

## Resumen

**Objetivo.:**

Comparar la eficacia de las visitas domiciliarias y del seguimiento telefónico sobre la autoeficacia de los pacientes sometidos a cirugía de derivación arterial coronaria con injerto (CABG en inglés) y la carga de sus cuidadores.

**Métodos.:**

En este ensayo clínico aleatorio, 114 pacientes sometidos a CABG se asignaron a uno de los tres grupos de investigación: visitas domiciliarias, seguimiento telefónico y control sobre la base de la aleatorización en bloque estratificada. El programa de educación en autoeficacia en el grupo de visitas domiciliarias incluía cuatro sesiones presenciales de formación de 60 minutos una vez a la semana, y para el grupo de seguimiento telefónico, cuatro sesiones de asesoramiento telefónico de 30 minutos dos veces por semana por un mes. El grupo de control recibió atención rutinaria. Los datos se recogieron mediante el cuestionario de autoeficacia de la rehabilitación cardíaca y la escala de carga del cuidador antes y después de la intervención.

**Resultados.:**

Antes del estudio, no hubo diferencias entre los tres grupos en cuanto a las medias de las puntuaciones de las escalas de autoeficacia y carga del cuidador. Sin embargo, se encontró una diferencia estadísticamente significativa entre los grupos de visitas domiciliarias y de control (*p*<0.001) y entre los grupos de seguimiento telefónico y de control (*p*<0.001) después de la intervención, con un aumento de la autoeficacia y una reducción de la carga del cuidador. Es de anotar que no hubo diferencias significativas entre los grupos de visita domiciliaria y de seguimiento telefónico en cuanto a las puntuaciones de autoeficacia y de carga del cuidador.

**Conclusión.:**

Ambos métodos de educación en este estudio tuvieron una efectividad similar en el aumento de la autoeficacia y la reducción de la carga de los cuidadores tras el alta de los pacientes sometidos a CABG.

## Resumo

**Objetivo.:**

Comparar a eficácia das visitas domiciliares e do seguimento telefónico sobre a autoeficácia dos pacientes submetidos a cirurgia de derivação arterial coronária com enxerto (CABG em inglês) e a carga dos seus cuidadores.

**Métodos.:**

Neste ensaio clínico aleatório, 114 pacientes submetidos a CABG foram designados a um dos três grupos de investigação: visitas domiciliares, seguimento telefónico e controle sobre a base aleatorizada em blocos estratificada. O programa de educação em autoeficácia no grupo de visitas domiciliares incluía quatro sessões de formação presenciais de 60 minutos uma vez por semana, e para o grupo de seguimento telefónico, quatro sessões de assessoramento telefónico de 30 minutos duas vezes por semana durante um mês. O grupo de controle recebeu atenção rotineira. Os dados se recolheram mediante o questionário de autoeficácia da reabilitação cardíaca e a escala de carga do cuidador antes e depois da intervenção.

**Resultados.:**

Antes do estudo, não houve diferenças entre os três grupos enquanto às médias das pontuações das escalas de autoeficácia e carga do cuidador. Porém, se encontrou uma diferença estatisticamente significativa entre os grupos de visitas domiciliares e de controle (*p*<0.001) e entre os grupos de seguimento telefónico e de controle (*p*<0.001) depois da intervenção, com um aumento da autoeficácia e uma redução da carga do cuidador. É importante anotar que não houve diferenças significativas entre os grupos de visita domiciliar e de seguimento telefónico enquanto às pontuações de autoeficácia e de carga do cuidador.

**Conclusão.:**

Ambos os métodos de educação neste estudo tiveram uma efetividade similar no aumento da autoeficácia e a redução da carga dos cuidadores após a alta dos pacientes submetidos a CABG.

## Introduction

Following the increasing prevalence of coronary artery disease and the resulting mortality, coronary artery bypass graft is one of the primary and most common surgical procedures in the treatment of cardiovascular diseases, such that about 8 million people undergo CABG globally each year, about 40 000 of which are performed in Iran.(1) The advantages of CABG include alleviation of symptoms, increased survival rates, and improvements in patient function، However, many physical, mental, and social problems may occur after the operation. These problems include shortness of breath, pain in the incision area, weakness, insomnia, fear, leg edema, wound infection, palpitations, and digestive problems.(2) A study reported that 69% of patients experienced shortness of breath, 39% sleep disorders, 39% incisional pain, and 18% anorexia three weeks after surgery.(3)

Since the continuation of these complications reduces the quality of life (QoL) of patients (2), reducing complications after open-heart surgery is an essential factor, and improving the self-efficacy of patients and caregivers can play a significant role in improving the condition of patients after surgery.(4) Self-efficacy is the belief that a person has performed an activity successfully.(5) Providing special education to patients and raising their awareness of risk factors(6) and the treatment and control of the disease leads to improved health, adherence to the recommended therapies, facilitation of healthy habits and behaviors, and adjustment of mental status, and thus increases their self-efficacy.(7) However, the results of many studies indicate that the self-efficacy of cardiac patients is weak to moderate.(8,9) One study reported poor self-efficacy in heart disease patients.(10) In addition to low self-efficacy, patients often rely on the family for self-care during recovery,(11) and most family caregivers are responsible for administering medications, facilitating communication, and continued emotional support. Caregivers, as individuals vulnerable to the mentioned problems and most notably responsible for managing financial issues, experience anxiety, depression, and concerns about the possibility of losing the patient, and may suffer from mood disorders and lose their courage. At the same time, their underlying diseases are ignored.(12)

Therefore, in order to maintain healthy behaviors, such as adherence to medications, quitting smoking, weight control, regular exercise, and a healthy diet, and as a result, to improve heart patients’ self-efficacy and to reduce the subsequent caregiver burden, there is a need to develop and implement self-management interventions.(13) Self-management is recognized as a method to help patients with chronic conditions enjoy the best possible quality of life.(14) The concept of self-management is derived from the cognitive learning theory and is defined as the ability of an individual to manage symptoms, treatment, physical and psychological outcomes, and to modify lifestyle in dealing with a chronic disease.(15) Self-management programs can increase patients’ awareness and ability to manage disease symptoms, perform self-care behaviors, and increase patients’ self-efficacy.(16) Home-based cardiac rehabilitation self-management programs, including monitoring and follow-up visits, are conducted in person, by telephone, or online with the help of healthcare professionals.(15) Homecare services are provided worldwide and have different meanings for different countries. This care method is provided during the recovery process and after the patient is discharged, especially during the first few weeks after discharge. The provision of home care services varies depending on the cultural context of each community.(17)

As one of the most critical components of home care, home visits have reduced patient readmissions by 25% and reduced treatment costs significantly.(18) Despite the effectiveness of homecare follow-ups, this method requires considerable quantities of human resources, time, and money. Therefore, using means of distance communication such as telephone calls to follow up on patients' homecare has been proposed to solve these problems.(8) Follow-up calls by nurses can be used as a valuable method for information exchange, symptom checks, rapid detection of complications, improvement of clinical condition, improvement of quality of life, and providing reassurance to the patient and his/her family.(19) However, while the use of home care services has been on the rise worldwide in recent years and despite advances in remote care, people generally have many problems with the use of smartphones, tablets, laptops, or computers, which are more pronounced in countries such as Iran.(20) In 2011, the American Heart Association announced that home-based rehabilitation could be a viable option for heart patients، and a study found that providing home-based services to patients with heart problems could improve their quality of life.(21)

However, this healthcare method has not been institutionalized in Iran, a developing Middle Eastern country, where the burden of chronic diseases and population aging is increasing.(22) Additionally, hospital services take precedence over community-based services in the Iranian health system, and there are barriers to the provision of homecare such as cultural issues, society’s negative perception about the role of nurses, lack of insurance coverage, safety and security issues, and lack of trust in nurses.(17) In many subspecialty cardiac hospitals in Iran, open-heart surgery is performed, but most hospitals do not have written plans to follow up on these patients in the community after discharge. Many patients develop various physical and psychological complications after discharge. The Shahid Madani Cardiac Hospital of the Lorestan Province of Iran, as the only subspecialty hospital in the province, provides services to 1760649 people. There is no post-discharge follow-up system in this province, and many patients suffer from various physical and mental complications after discharge.

On the other hand, the wide geographical distribution of patients’ residences necessitates some services performed in absentia. It seems that providing community-based services can alleviate some of the patients' problems. Given that no studies were found to have evaluated the effectiveness of different community-based care methods for open-heart surgery patients, this study aimed to compare the effectiveness of home visits and follow-up calls on the self-efficacy of patients who had undergone CABG and the burden of family caregivers.

## Methods

This pretest-posttest randomized controlled trial was conducted from July 2018 to August 2019 in one of the Lorestan University of Medical Sciences hospitals. First, 205 people were selected, and after reviewing their medical records and considering the inclusion criteria, 120 patients were enrolled in the study. With a dropout rate of 5%, 114 patients who had undergone CABG and had been discharged from the open-heart surgery intensive care unit completed the study.

Inclusion criteria were willingness to participate in the study, a history of coronary artery bypass surgery (under cardiopulmonary pump), an age range of 30 to 70 years, number of grafts (three, four, or five), access to a landline or mobile phones, and living within a 90 km maximum radius from the city of Khorramabad. Exclusion criteria included unwillingness to participate in the study for any reason, previous history of participation in cardiac rehabilitation programs, presence of any known psychiatric or psychomotor disorders, suffering from any hearing, vision, and speech disorders, suffering from chronic debilitating diseases, readmission, death of the patient, and researchers’ inability to reach the patient and the occurrence of acute physical or mental disorders in the patient. For caregivers, inclusion criteria were willingness to participate in the study, direct caregiver, literate, 18 years old and older. The exclusion criteria for caregivers were unwillingness to continue cooperation with the researchers, the caregiver’s unwillingness to continue caring for the patient, having restrictive neurological or motor diseases, and having a background in medical sciences.

Then, based on block randomization, the participants were placed in one of three groups in the self-management program: follow-up calls, home visits, and control. Thus, we had 20 blocks with a six-letter arrangement, including two A letters, two B letters, and two C letters. group A received home visits, group B was the follow-up calls group, and group C was the control group. The individuals were entered into each group in the order of the selected blocks using the arrangement inside the blocks at random by a researcher colleague who was unaware of the groups. The sample size was determined for each group as 40 people based on comparing the two means, based on similar studies on self-efficacy.(23)

**Figure 1:**
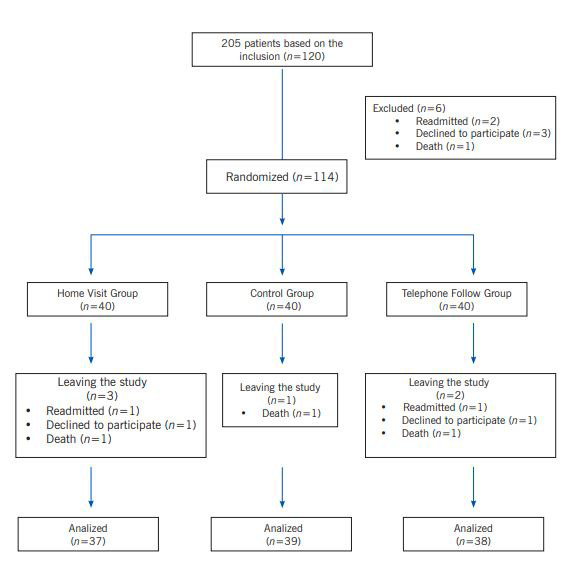
Classification, allocation, and analysis of participant data

Of the 120 patients initially admitted to the study, six were excluded for some reason. The final number of participants for each group was 38 for the Telephone follow-up group), 37 for the home visits group, and 39 for the control group (Figure 1). Individuals were randomized into each group in the order of the letter of the selected blocks using the internal arrangement of the block. The number of strata of the blocks was 20, and the arrangement inside each block consisted of 6 participants, including two participants for each study group. The home visits group was designated with the letter A, the telephone follow-up group was designated with the letter B, and the letter C was assigned to the control group. The self-management program was followed up on both intervention groups one day after discharge and usually starting at four PM. One of the researchers (the first author), a nurse in the Cardiac Surgery Intensive Care Unit with about ten years of experience in this department, answered patients’ questions from 8:00 AM to 11:00 PM. At the beginning of each session before starting the follow-up intervention, an initial assessment of the patient regarding the amount of information the patient had and the desire to learn, and an estimation of the patient’s educational needs was carried out, and the necessary interventions and training were performed based on the results of the initial assessment.

The self-management program focused on education on issues such as 1. background information on heart disease and surgery, 2. how to exercise and stay active, sleep, rest, and daily activities, 3. diet, 4. the prescribed medications, 5. risk factor management, 6. observance of personal health, 7. how to take care of surgical wounds, and 8. follow-up and subsequent referrals and psychological counseling and interventions with the help of a psychologist, if necessary. In order to organize the training, an educational booklet was used, the content of which was prepared by reviewing authoritative textbooks, articles, and guidelines and considering the needs of CABG patients. The qualitative comments of 8 specialists, including three nursing faculty members, a cardiovascular surgeon, two cardiovascular nurses, a nutritionist, and a psychologist, were used to verify the content validity of the booklet.

The Home Visit Group. The self-management program was conducted for the home visit group with a nurse visiting the patient at home and assessing his/her information needs about self-management programs and by offering face-to-face training to the patient with the participation of the home caregiver for four weeks (one session a week, four sessions in all), each session lasting for about one hour. In the first week of the intervention, general information about taking medications, wound care, types of allowed activity, exercise and taking slow walks, active and passive exercises, diet, individual health, respiratory exercises, and awareness of postoperative complications and symptoms was provided the patients.

The Telephone Follow-up Group. In the telephone follow-up group, telephone counseling was performed twice a week, in eight 30-minute sessions. The call times were selected with prior coordination with and consent of the patient and his/her family. The different dimensions of the intervention (learning materials and contents, goals) for this group were similar to the home visit group, except for the follow-up method. Thus, telephone counseling and oral explanations offered the telephone follow-up group training, but in the home visit group, the activities were carried out in the researcher's presence and under his supervision and training. In addition, booklets were provided to the patients and their families that illustrated how the exercises should be performed.

Training about staying active in both group: In the first and second weeks, patients’ mobility started with limited and low-intensity exercises according to the patient’s tolerance ranging from getting out of bed and walking to active exercises of the wrist, elbow, and ankle operated upon. In the third and fourth weeks, mobility training continued with active upper and lower limb exercises. First, stretching movements were taught in the form of slow rotational movements of the neck, shoulders, ankle movements within the range of motion of the joints, and then the exercises of the upper limbs were taught, for instance, raising the arms above the head and then lowering them. For knee exercise, the patient was instructed to sit on a chair and, in turn, press each leg’s toes on the ground and then raise the leg so that the calf and the thigh forms a straight line and the toes are bent towards the abdomen to the extent that the calf muscles contract. Finally, aerobic exercises and jogging were recommended and performed. The patient and the caregiver were instructed to repeat all these exercises 5 to 6 times daily, and each move is repeated ten times in each exercise session.

The structure and content of the self-management program are shown in Table 1.

**Table 1:** The structure and contents of the self-management program

Groups	Sessions	Topics	Description of the Topics	Time (Minutes)
Home visits	1	General explanations on care, administration of medications, physical activity, and answering the raised questions	The nurse answered all questions and, ambiguities were resolved. General information about primary care, complications, and emergencies, referrals to the doctor, how to take the drugs, side effects, and precautions about drugs were presented orally and, if necessary, demonstrated in practice. Additionally, permitted physical activities were taught to the patients and caregivers.	60
Home visits	2	Respiratory care, physical mobility, rest and sleep, physical activity, and answering the questions	Breathing exercises, using a Spiro-Ball, the use of respiratory sprays, the time to get out of bed, the precautions that should be taken before and during getting out of bed, the amount of rest, shortness of breath and its care, and the allowed physical activities were demonstrated to the patients and caregivers in practice.	60
Home visits	3	Wound care, personal hygiene, physical activities, and answering the raised questions	How to change wound dressings, signs of wound infection, washing, and bathing, the time to remove the sutures, put on clothes, and clean and disinfect them were taught and demonstrated to the patients and caregivers.	60
Home visits	4	Nutrition, exercise, physical activities, and answering the questions	The proper diet, the number of meals, the time of meals, the proper amount of food to be taken, methods of exercising and proper times for it, and the recommended physical activities were taught and demonstrated to the patients and caregivers under monitoring a nurse.	60
Telephone follow-up	1	General information about types of care, physical activities, Answering the questions raised by patients and caregivers.	All the questions raised by patients were answered, and general information on primary care, emergencies, visits to doctors, and the permitted physical activities were taught to the patients and caregivers by phone.	30
Telephone follow-up	2	have taken medications, physical activity, and answered the questions.	Taking the medications, and giving information about drug side effects, appropriate care, precautions, and the permitted physical activities were explained by phone to the patients and caregivers.	30
Telephone follow-up	3	Respiratory care, teaching physical activities, and answering the questions.	Breathing exercises and the use of Spiro-Ball, shortness of breath, the appropriate care, the methods of using respiratory sprays, and the allowed physical activities were discussed on the phone with patients and caregivers.	30
Telephone follow-up	4	Staying active, resting, sleeping, doing physical activities, and answering the questions.	Teach patients how and when to get out of bed, the precautions that should be taken before and during getting out of bed, the amount of rest needed, and the permitted physical activities on the phone.	30
Telephone follow-up	5	Wound care and answering the questions.	Patients and caregivers were taught how to change wound dressings, the signs of wound infection, and remove the sutures by phone.	30
Telephone follow-up	6	Nutrition and answering questions.	Patients and caregivers were taught about the proper diet, the number of meals, the time of having the meals by phone.	30
Telephone follow-up	7	Personal hygiene Answering the questions.	Patients and caregivers were taught about washing, bathing, putting on clothes, and cleanliness by phone.	30
Telephone follow-up	8	Physical exercise and answering questions.	The patients and caregivers were taught different types of physical exercise and the permitted times and amounts.	30

The Control Group. This group also included routine care, including patient education with the participation of a home caregiver on how to exercise at home, the administration of medications, personal health, changing wound dressings for follow-up visits, and diet according to the routine of the relevant medical center. A nurse provided the education orally and face-to-face with the hospital’s educational booklet during the discharge process.

Research Instruments. The cardiac rehabilitation self-efficacy instrument developed by Jokar *et al*.(24) was administered on the first day and the last day of the fourth week after discharge. The first part of the instrument consisted of items about the demographic characteristics of the patients, including age, gender, marital status, education, place of residence, occupation, economic status, the reason for referral, the number of sessions attended, the prescribed medications, home address, and phone number. The second part comprised 55 items rated on a 5-point Likert scale from 0 = “I am not sure” to 4 = “I am completely certain.” On this instrument, the possible scores range from 0 to 220, 0 indicating the minimum and 220 indicating the maximum cardiac rehabilitation self-efficacy score. Obtaining a score from 0 to 55, 56 to 110, 111 to 165, 166 to 220 indicated low, moderate, good, and very good cardiac rehabilitation self-efficacy, respectively. The targeting instrument was locally designed and psychometrically evaluated based on proper principles. Internal consistency was used to test and confirm the instrument’s reliability with a Cronbach’s alpha of 0.92. The instrument's validity was verified by confirmatory and exploratory factor analysis.(9) 

Novak and Guest’s caregiver burden inventory (CBI) consisted of two parts, completed by patients’ main caregivers. The first part collects the demographic information. The second part comprises 24 items with five subscales: time-dependent caregiver burden, developmental caregiver burden, physical caregiver burden, social caregiver burden, and emotional caregiver burden. Caregivers’ answers were indicated on a 5-point Likert scale from “completely incorrect” to “completely correct.” The participants are expected to select one of these options in answering the questions: 1 = “completely incorrect”, 2 = “incorrect”, 3 = “partly correct”, 4 = “correct”, 5 = “completely correct”. The scores for the whole scale range from 24 to 120. Scores from 24 to 39 indicate mild caregiver burden, 40 to 71 suggest moderate caregiver burden, and 72 t0 120 indicate intense care burden. The reliability and validity of this instrument are acceptable. The opinions of several experts to validate the instrument were sought. The reliability was confirmed with Cronbach’s alphas of 69-87 for the subscales and 80 for the whole scale.(25)

The demographic information was collected by interviewing the patients and caregivers and referring to their clinical medical records. CBI was first completed for all groups on the first day after discharge. In the home visits group, the instrument was completed by patients and caregivers at their homes during the last session. In the other two groups, CBI was completed on the last day of the fourth week after discharge in person by a researcher who visited their homes. Additionally, all the topics were taught theoretically and practically to patients and caregivers of these groups during this last home visit. Both the home visits group and the follow-up calls group members were assured that the researcher would answer any questions they might have during the study course, and the control group members were assured that they would receive the routine care offered by the relevant center.

Statistical Analysis. Statistical data analysis was performed by the descriptive statistical tests of mean and standard deviation and the analytical statistical tests of chi-square, variance analysis, and covariance analysis. The chi-squared test was used to compare the demographic information and the qualitative data of the groups. Analysis of variance was used to evaluate and compare quantitative demographic and clinical information and compare the groups’ self-efficacy scores before the intervention. Analysis of covariance was utilized to compare the self-efficacy scores of the groups after adjusting for the effects of confounding variables. The significance level was set at 0.05.

Ethical Considerations. Before starting the study, ethical approval of the Vice Chancellorship for Research and Technology of the Lorestan University of Medical Sciences (ethics code: LUMS.REC.1397.027). This study was conducted under the Declaration of Helsinki. The study’s objectives were explained to the patients, and informed consent was obtained from them for participation in the study without any form of compulsion. Moreover, all topics taught to the intervention groups were also taught to the control group in the last session. The trial registration number was IRCT20100609004129N2.

## Results

114 patients with an average age of 60.43±6.17 were enrolled in the study. Most participants were male (65.79%), married (92%), illiterate (55.3%), and urban residents (79.82%). No significant differences were observed among the three groups regarding demographic and clinical characteristics, including age, gender, occupation, residence, the number of grafts, chronic diseases, smoking, and sleep disorders (Table 2).

**Table 2:** Comparing the demographic information of the three groups of CABG surgery patients

Group	Home visits *n*=38	Home visits *n*=38	Follow-up calls *n*=39	Control *n*=37	***p*-value**
Variable	***n* (%)**	***n* (%)**	***n* (%)**	***n* (%)**	***p*-value**
Gender	Male	23 (62.2%)	29 (74.7%)	23 (60.5%)	0.378
Gender	Female	14 (37.8%)	109 (25.6%)	15 (39.5%)	0.378
Marital Status	Married	35 (94.6%)	35 (89.7%)	35 (92.1%)	0.418
Marital Status	Single	2 (5.4%)	4 (10.3%)	3 (7.9%)	0.418
Place of residence	Urban areas	33 (89.2%)	28 (71.8%)	30 (78.9%)	0.325
Place of residence	Rural areas	4 (10.8%)	11 (28.2%)	8 (21.1%)	0.325
Education	Illiterate	22 (59.5%)	16 (41%)	25 (65.8%)	0.270
Education	Junior high	3 (8.1%)	12 (30.8%)	5 (13.2%)	0.270
Education	High school	3 (8.1%)	2 (5.1%)	2 (5.3%)	0.270
Education	High school diploma	5 (13.5%)	6 (15.4%)	4 (10.5%)	0.270
Education	University	4 (10.8%)	3 (7.7%)	2 (5.3%)	0.270
Number of children	0 to 3	11 (29.7%)	13 (33.3%)	10 (26.3%)	0.978
Number of children	4 to 6	21 (58.8%)	14 (35.9%)	13 (34.2%)	0.978
Number of children	More than 6	5 (13.5%)	12 (30.8%)	15 (39.5%)	0.978
The number of bypass grafts	3	10 (27%)	7 (17.9%)	9 (23.7%)	0.495
The number of bypass grafts	4	22 (59.5%)	29 (74.4%)	22 (57.9%)	0.495
The number of bypass grafts	5	5 (13.5%)	3 (7.7%)	7 (18.4%)	0.495
Does the patient suffer from a sleep disorder?	Yes	9 (24.3%)	7 (17.9%)	10 (26.3%)	0.657
Does the patient suffer from a sleep disorder?	No	28 (75.7%)	32 (82.1%)	28 (73.7%)	0.657
Cardiac ejection fraction	Normal	24 (64.9%)	21 (53.8%)	21 (55.3%)	0.575
Cardiac ejection fraction	Abnormal	13 (35.1%)	18 (46.2%)	17 (44.7%)	0.575
Is the patient a smoker?	Yes	20 (51.4%)	22 (56.4%)	22 (57.9%)	0.945
Is the patient a smoker?	No	17 (49.5%)	17 (43.6%)	16 (42.1%)	0.945
Does the patient have a history of blood pressure?	Yes	35 (94.6%)	34 (87.2%)	37 (97.4%)	0.0194
Does the patient have a history of blood pressure?	No	2 (5.4%)	5 (12.8%)	1 (2.6%)	0.0194
Does the patient have a history of thyroid disorders?	Yes	2 (5.4%)	1 (2.6%)	295.3%)	0.791
Does the patient have a history of thyroid disorders?	No	35 (94.6%)	38 (97.4%)	36 (94.7%)	0.791
Does the patient have a history of digestive disorders?	Yes	6 (16.2%)	4 (10.3%)	5 (13.2%)	0.744
Does the patient have a history of digestive disorders?	No	31 (83.8%)	35 (89.7%)	33 (86.8%)	0.744
Does the patient have a history of diabetes?	Yes	9 (24.3%)	14 (35.9%)	13 (34.2%)	0.507
Does the patient have a history of diabetes?	No	28 (75.7%)	25 (64.1%)	25 (65.8%)	0.507
Does the patient have a history of musculoskeletal disorders?	Yes	1 (2.7%)	1 (2.6%)	0 (0%)	0.601
Does the patient have a history of musculoskeletal disorders?	No	36 (97.3%)	38 (97.4%)	38 (100%)	0.601
Does the patient have a history of dyslipidemia?	Yes	4 (10.8%)	10 (25.6%)	8 (21.1%)	0.247
Does the patient have a history of dyslipidemia?	No	33 (89.2%)	29 (74.4%)	30 (78.9%)	0.247
Does the patient have a history of COPD?	Yes	29 (78.4%)	29 (74.4%)	32 (84.2%)	0.567
Does the patient have a history of COPD?	No	8 (21.6%)	10 (25.6%)	6 (15.8%)	0.567
Does the patient have a history of CVA?	Yes	34 (91.9%)	34 (87.2%)	35 (92.1%)	0.710
Does the patient have a history of CVA?	No	3 (8.1%)	5 (12.8%)	3 (7.9%)	0.710
Does the patient have a family history of CVD?	Yes	14 (937.8%)	18 (46.2%)	12 (31.6%0	0.419
Does the patient have a family history of CVD?	No	23 (62.2%)	21 (53.8%)	23 (68.4%)	0.419

Based on a one-way analysis of variance, the three groups were not significantly different before the intervention in terms of self-efficacy (*p* = 0.960). However, the results of covariance analysis showed significant differences among the groups after the intervention (*p* = 0.001), such that the mean self-efficacy scores in the home visits and follow-up calls groups were higher than the control group (*p* = 0.001). However, there were no significant differences between the home visits and follow-up calls groups after the intervention (*p* = 0.477) (Table 3). Bonferroni’s posthoc test showed significant differences in self-efficacy scores between the home visits and control groups (*p* = 0.001) and the follow-up calls and control groups (*p* = 0.001) after the intervention.

**Table 3:** The mean self-efficacy score of CABG patients and their significance level in groups before and after the intervention

Groups	Groups	Mean (SD)	F*	df	***p*-value**
Before	Home visits	131.40 (22.82)	0.041*	2	0.960
Before	Follow-up calls	132.94 (31.03)	0.041*	2	0.960
Before	Control	131.43 (26.56)	0.041*	2	0.960
After	Home visits	175.81 (24.723)	19.73**	2	0.001
After	Follow-up calls	179.79 (30.61)	19.73**	2	0.001
After	Control	147.37 (31.44)	19.73**	2	0.001

* One-way analysis of variance, ** analysis of covariance

Considering Table 4 and the results of the chi-squared test, there were no significant differences among the three groups in terms of the demographic variables of gender (p = 0.794), marital status (*p* = 0.652), education (*p* = 0.497), occupational class (*p* = 0.302), and the relationship between the patient and the caregiver (*p* = 0.531). According to Table 5, the caregiver burden was significantly different in the three groups after the intervention (*p* = 0.001). Bonferroni’s posthoc test showed that the caregiver burden was significantly different in the home visits and control groups (p = 0.001), as well as in the follow-up calls and control groups (*p* = 0.001) after the intervention, such that the mean caregiver burden scores were less than in the control group. However, no significant difference was found between the home visits’ and follow-up calls groups (*p* = 0.801). Analysis of covariance was used to evaluate the differences among the three groups after the intervention (Table 5).

**Table 4:** The frequency of the demographic characteristics of caregivers

Group	**Home visits *n*=38**	**Follow-up calls *n*=39**	**Control *n*=37**	***p*-value**
Variable	***n* (%)**	***n* (%)**	***n* (%)**	***p*-value**
Gender	6 (16.2%)	5 (12.8%)	7 (18.4%)	0.794
Gender	31 (83.8%)	34 (87.2%)	31 (81.6%)	0.794
Marital status	10 (27%)	12 (30.8%)	13 (34.2%)	0.652
Marital status	27 (73%)	27 (69.2%)	25 (65.8%)	0.652
Education	1 (2.7%)	4 (10.2%)	1 (2.6%)	0.497
Education	3 (8.2%)	6 (15.4%)	8 (21.1%)	0.497
Education	7 (18.9%)	5 (12.8%)	4 (10.5%)	0.497
Education	17 (45.9%)	18 (46.2%)	15 (39.5%)	0.497
Education	9 (24.3%0	6 (15.4%)	10 (26.3%)	0.497
Job classification	0 (0%)	0 (0%)	2 (5.3%)	0.302
Job classification	0 (0%)	3 (7.7%)	3 (7.9%)	0.302
Job classification	6 (16.2%0	5 (12.8%)	9 (23.7%)	0.302
Job classification	25 (67.6%)	25 (64.1%)	20 (52.6%)	0.302
Job classification	6 (16.2%0	6 (15.4%)	4 (10.5%)	0.302
Caregiver and patient relationship	12 (32.4%)	15 (35.8%)	9 (23.7%)	0.531
Caregiver and patient relationship	21 (56.8%)	19 (48.7%)	22 (57.9%)	0.531
Caregiver and patient relationship	4 (10.8%)	4 (10.3%)	7 (18.4%)	0.531
Caregiver and patient relationship	0 (0%)	1 (2.5%)	0 (0%)	0.531

**Table 5:** The comparison of the caregiver burden of the three groups before and after the intervention

Groups	Mean (SD) of caregiver burden	F	df	***p*-value**
Before	Before	Before	Before	Before
Home visits	(13.50) 58.83	0.229*	2	
Telephone follow-up	(16.53) 60.64	0.229*	2	
Control	(11.39) 60.81	0.229*	2	
After	After	After	After	After
Home visits	(8.04) 34.10	21.96**	2	0.001
Telephone follow-up	(8.836) 34.58	21.96**	2	0.001
Control	(14.03) 48.23	21.96**	2	0.001

* One-way analysis of variance; * Analysis of covariance.

## Discussion

The present study’s findings indicated that the self-efficacy of CABG surgery patients increased in the home visits and follow-up calls groups after the designed intervention was implemented compared to the control group in which the scores remained the same. In addition, the caregiver burden decreased in these groups after the one-month intervention was executed. In line with the present study, other studies have also shown that homecare for cardiac patients can improve self-efficacy and the quality of life (Qol).(7,8,26) Enhancing self-efficacy can improve self-care activities and health-related behaviors(27) and effectively reduce postoperative complications. In this study, the services were provided by a nurse with expertise in cardiac surgery patients. However, a health team should provide the required services.(17) Studies have also shown that providing services to cardiovascular patients by a multidisciplinary team under the guidance of nurses, physicians, nutritionists, and physiotherapists has led to improvements in self-management behaviors and self-efficacy.(14)

The present study demonstrated that telephone-based educational interventions could promote self-efficacy. Consistent with the present study, the findings of another study indicate that persistent follow-up sessions carried out in absentia or remotely involving an educational program with a duration of 3 months and presenting educational content via text messages and telephone calls can lead to increases in self-efficacy the patients.(6) Additionally, long-distance healthcare (telehealth) can increase QoL and decrease depression, anxiety, and suicide rates.(28) One study has shown that a one-unit increase in health-related behaviors and heart-related knowledge can raise self-efficacy in managing the prevention of cardiovascular diseases by 0.432 and 0.475 units, respectively.(29)

In the current study, communication with the patients and caregivers was conducted only by phone, whereas another study demonstrated that video calls might improve patients’ conditions after discharge.(20) However, another study suggested that implementing educational interventions using various media is more effective in enhancing patients’ self-efficacy than using telephones alone.(30) Another study, aiming to examine the effect of patients’ adherence to care programs, in which CABG patients had participated for four weeks and had been educated by video conferencing, showed that patients who had received services by telenursing were more successful in adhering to the treatment program than the control group.(20) Accordingly, it seems that using the potentials of social messaging apps may be more effective than phone calls. Therefore, it is recommended that a study be conducted on the impact of various methods of communication that might be used in telenursing in remote follow-up programs carried out in absentia. Nonetheless, another study has shown that this type of intervention is not financially cost-effective, the reason being the costs of digital instruments for hemodynamic indicators and weighing patients in absentia and the transfer of information via landlines.(31) The findings of this study showed that follow-up calls could eliminate distance and facilitate educating patients and caregivers.

Similar studies confirm that implementing self-management programs via various means and media can improve patients’ self-efficacy. A study demonstrated that group education, home visits to offer counsel, and follow-up calls could improve patients’ QoL and decrease postoperative problems. In this study, patients benefited both from home visits and follow-up calls. Considering the effectiveness of both rehabilitation methods, the simultaneous use of both methods can have a potentiating effect and the subsequent positive effects of rehabilitation.(26) Therefore, given the effectiveness of both methods and the cost-effectiveness of telephone counseling, and also because of lack of time and distance constraints, the lack of need to take trips and incur additional costs, the use of this method is recommended, especially in the clinical context of Iran, because answering patients’ questions regarding lifestyle choices and other issues related to patient rehabilitation by telenursing, as a system for offering health services to patients after surgery and discharge, can be beneficial in improving adherence to the treatment plan and improving QoL.

Moreover, the present study’s findings indicated that caregiver burden decreased in the home visits and follow-up calls groups, which is in line with a study by Nosratabadi *et al*.(22) Li Chi *et al*.(3) conducted a study aiming to reduce the family member caregiver burden of patients suffering from heart failure by phone demonstrated that intervention group caregivers improved in stress management and performance compared to the control group. Cardiovascular diseases accompany patients throughout their lives and affect other family members, too. Therefore, empowering the family to manage the disease better and enjoy life is essential.(32) In this regard, the findings of a study showed that even secondary caregivers suffer from physical and psychological problems during the provision of care services and need to be supported.(33) Although the challenges and problems that family caregivers face decrease over time due to gaining experience,(34) conducting patient-oriented interventions, including educational interventions, as in the present study, are essential in reducing the care burden of family caregivers.(23)

One of the limitations of this study was that it was conducted in a short period. On the other hand, multidisciplinary teams should make home visits and improve by inter-professional cooperation. In the present study, a team did not provide the services because of limitations. Hence, it is recommended that further studies be conducted on providing community-based services to patients after open-heart surgeries with more extended follow-up periods and team services. Another limitation of the study was the short duration of the educational intervention and the counseling offered to patients and their families.

## Conclusion

Based on the findings that showed both the home visits and the follow-up calls methods are effective, and there are no differences between them in results, it is suggested that both methods be used in order to improve the quality of health and treatment services after patients are discharged, such that home visits are made during the first days after discharge and then considering the condition of the patient and the family, follow-up calls are used subsequently. Furthermore, it is recommended that further studies be conducted on the impact of these methods on patients’ QoL, patient and family satisfaction, and also their cost-effectiveness of these methods at the community level.
